# A review of roll-to-roll nanoimprint lithography

**DOI:** 10.1186/1556-276X-9-320

**Published:** 2014-06-25

**Authors:** Nazrin Kooy, Khairudin Mohamed, Lee Tze Pin, Ooi Su Guan

**Affiliations:** 1 Nanofabrication and Functional Materials Research Group, School of Mechanical Engineering, Universiti Sains Malaysia, Engineering Campus, Nibong Tebal, Penang 14300, Malaysia

**Keywords:** Nanoimprint, Lithography, Nanofabrication, Nanopatterning, Roll-to-roll, Roll-to-plate, Plate-to-plate

## Abstract

Since its introduction in 1995, nanoimprint lithography has been demonstrated in many researches as a simple, low-cost, and high-throughput process for replicating micro- and nanoscale patterns. Due to its advantages, the nanoimprint lithography method has been rapidly developed over the years as a promising alternative to conventional nanolithography processes to fulfill the demands generated from the recent developments in the semiconductor and flexible electronics industries, which results in variations of the process. Roll-to-roll (R2R) nanoimprint lithography (NIL) is the most demanded technique due to its high-throughput fulfilling industrial-scale application. In the present work, a general literature review on the various types of nanoimprint lithography processes especially R2R NIL and the methods commonly adapted to fabricate imprint molds are presented to provide a clear view and understanding on the nanoimprint lithography technique as well as its recent developments.

**PACS:**

81.16.Nd

## Review

### Introduction

Recent developments in semiconductor and flexible electronics applications have observed a rapid increase in demands for lower cost, higher throughput, and higher resolution micro/nanofabrication techniques. This is due to the fact that conventional techniques such as electron beam lithography (EBL) have a low throughput [1] for mass production and other alternatives such as extreme ultraviolet lithography and focused ion beam lithography are very costly, limiting the technology only to large organizations [2].

Nanoimprint lithography (NIL) was introduced by Prof. S.Y. Chou and the team in 1995 [3] as a simpler, low-cost, and high-throughput alternative to micro- and nanofabrication. In the NIL process, a prefabricated mold containing an inverse of the desired patterns is pressed onto a resist-coated substrate to replicate the patterns via mechanical deformation. Hence, many replications may be produced from a single prefabricated mold using this method. As the NIL process is based on direct mechanical deformation, its resolution is not constrained to the limitations of light diffraction or beam scattering factors as observed in conventional nanolithography methods [4]. In terms of patterning capability, various 2D and 3D structures [5] with feature sizes ranging from several micrometers [6,7] down to sub-50-nm scale [8-10] have been demonstrated. Due to its promising potential, the NIL process has been added into the International Technology Roadmap for Semiconductors (ITRS) for 32- and 22-nm nodes [11] and has been widely researched and improvised by many researchers ever since, resulting in several variations of the process.

### Variant of nanoimprint lithography

#### ***NIL variants based on resist curing***

**Figure 1:**
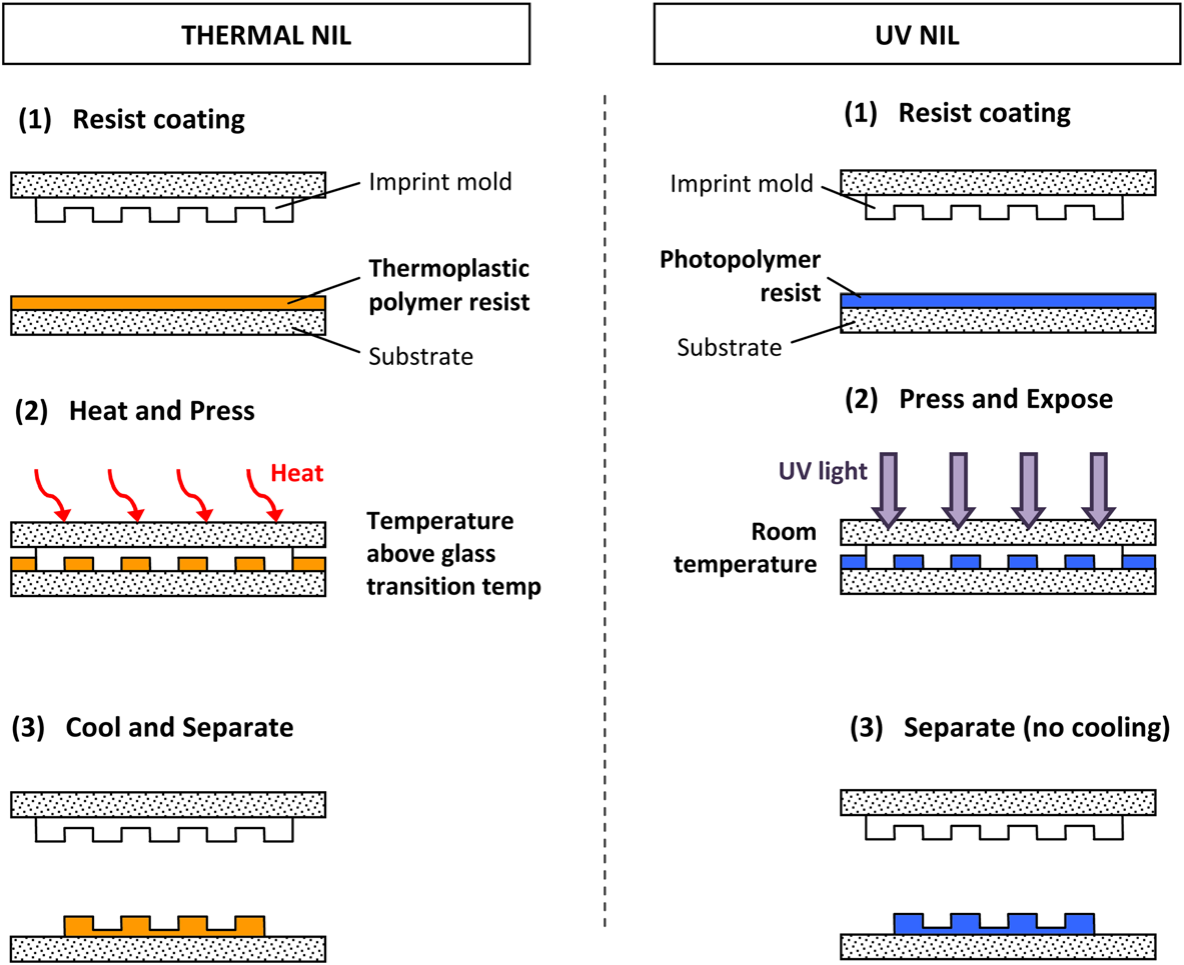
**A comparison of a typical thermal NIL **[3]** and UV NIL process **[5]**.**

In terms of resist curing, there are two fundamental types of the process: thermal NIL and ultraviolet (UV) NIL. The thermal NIL (also known as hot embossing) process is the earliest type of NIL introduced by Prof. S.Y. Chou [3], which involves imprinting onto a thermally softened thermoplastic polymer resist. A typical thermal NIL process is as follows: A mold is first heated up to an elevated temperature higher than the glass transition temperature (*T*_g_) of the thermoplastic polymer resist used. As the heated mold comes in contact with the resist, the resist will be heated up and soften into a molten stage, where it will fill in the mold cavities under sufficient imprinting pressure and time. The elevation of temperature is necessary because the elastic modulus and yield strength of the resin decreased considerably when the temperature exceeded *T*_g_. However, temperatures much higher than *T*_g_ can cause serious damage to the film [12]. The imprint temperature will then be lowered below the *T*_g_ of the resist to solidify the resist, before the mold is lifted. As a result, the patterns/structures from the mold are transferred to the resist. An illustration of a typical thermal NIL process is shown in Figure 1.

In contrary to the thermal NIL process, the UV NIL process involves imprinting onto a layer of liquid photopolymer resist and curing using UV exposure, which causes resist hardening due to cross-linking in the polymer instead of manipulating the phase change via resist temperature [13]. The remaining imprint mechanism, however, is similar to the thermal NIL process. A typical UV NIL process is also illustrated in Figure 1 for comparison purposes. The UV NIL process has several prominent advantages over the thermal NIL process, which include the capability of UV NIL to be conducted at room temperature without the need of elevated temperature imprinting [5,14], which helps eliminate the issues resulting from thermal expansion variations between the mold, substrate, and resist. In addition, the imprinting process involves a less viscous liquid photoresist, which allows the process to be conducted at a lower imprint pressure compared to thermal NIL processes [11,14-16]. The lower viscosity of the resist also allows the resist to fill in the mold cavity in a shorter time, and the elimination of the temperature cycle also improves the process throughput [14].

**Figure 2:**
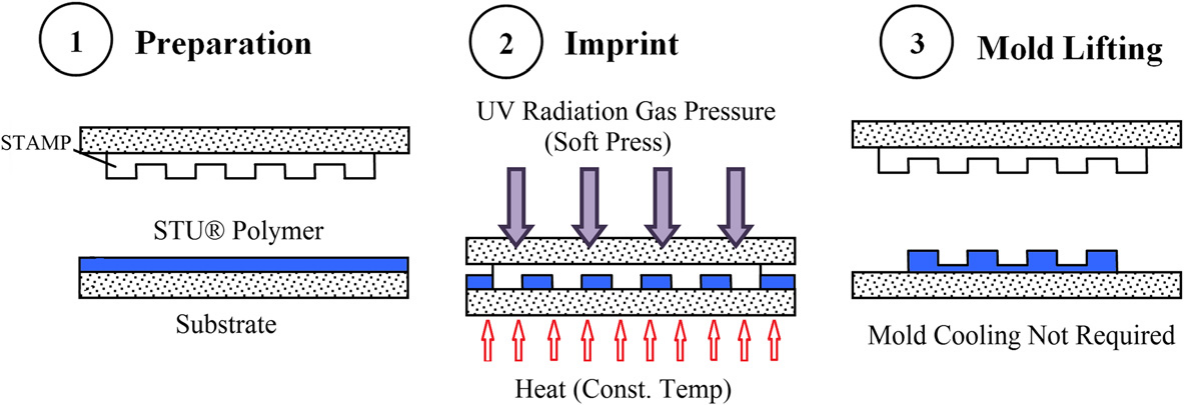
**Concept of the Simultaneous Thermal and UV (STU^®^) NIL process **[11]**.**

Besides the two fundamental processes, there are several other variants of NIL processes in terms of resist curing. The Simultaneous Thermal and UV (STU^®^) technology introduced by Obducat (Lund, Sweden) [11,17] allows a complete NIL cycle to be conducted at a constant temperature using both heating and UV exposure simultaneously on a UV-curable thermoplastic pre-polymer resist as shown in Figure 2. During the imprinting process, the applied heat helps soften the STU^®^ resist, which forms as a solid film at a temperature below its glass transition temperature, whereas the UV exposure solidifies the resist via polymer cross-linking. Besides eliminating the need for cooling time prior to mold lifting, the unique STU^®^ technology also helps in minimizing issues related to thermal expansion differences [18].

In addition, Chou and the team [19] also introduced the usage of a single XeCl excimer laser pulse to melt a thin layer up to 300 nm of the silicon substrate surface, where the molten silicon layer will then be imprinted using the mold. This NIL process is named laser-assisted direct imprint (LADI). Similar to thermal NIL in concept, the molten silicon layer will fill in the mold cavity under suitable imprinting pressure, transferring the patterns to the silicon substrate. The embossing time is reported to be less than 250 ns. A similar concept is also observed in [20], where a CO_2_ infrared laser is used to soften a thermoplastic resist for the NIL process.

#### ***NIL variants based on imprint contact***

**Figure 3:**
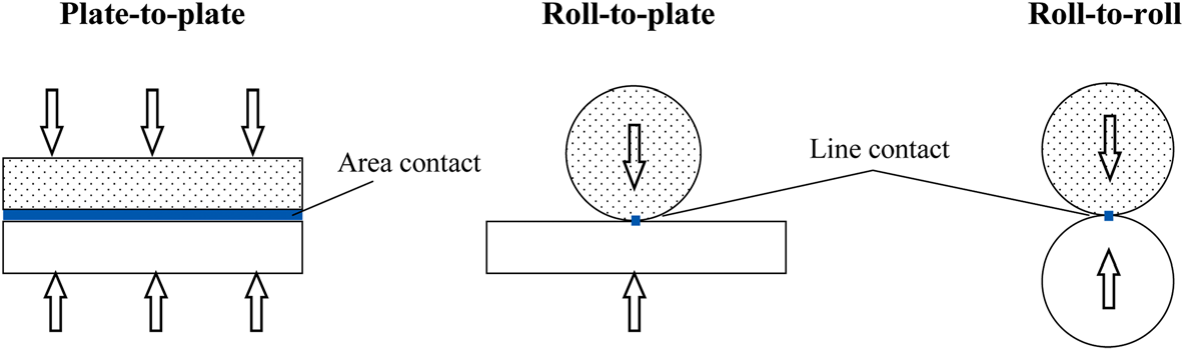
Three main contact types of NIL processes.

In terms of imprint contact types, NIL processes can be categorized into three types: plate-to-plate (P2P) NIL, roll-to-plate (R2P) NIL, and roll-to-roll (R2R) NIL. An illustration of each contact type is shown in Figure 3.

##### 

**Plate-to-plate NIL** In P2P NIL, a rigid flat stamp/mold (typically a patterned wafer) is used to imprint onto a resist layer on a flat rigid substrate, resulting in an area contact [3-5]. In general, P2P NIL may be conducted in two manners: single-step imprinting and multiple-step imprinting [11]. In single-step imprinting, the entire imprint area (usually the entire wafer) is imprinted in a single imprinting cycle regardless of its size. However, this method is typically unsuitable for large imprinting areas as it would require larger forces to provide a suitable imprint pressure, which may reach 20 kN of force for an 8-in. wafer [21]. Table 1 shows the imprint force used for P2P NIL processes in several research publications.

**Table 1:** Imprint forces used in P2P NIL processes from research publications for several different imprint areas

**Researcher**	**Imprint area**	**Imprint force (N)**
Lebib et al. [22]	8 mm × 8 mm	32 to 192
Chou et al. [8]	15 mm × 18 mm	1,116 to 3,537
Shinohara et al. [23]	27.4-mm diameter disc	3,000
Beck et al. [24]	2-in. wafer	14,188
Lebib et al. [25]	4-in. wafer	40,536
Perret et al. [21]	8-in. wafer	20,000

Additionally, air bubble entrapment issues are also commonly observed in P2P NIL, particularly in large-area, single-step processes [21,26] as air is easily trapped in the gaps between resist and mold cavities, resulting in defects on the imprinted structures. The risk of defects is increased when the mold contains depressions or when the resist is deposited as droplets rather than spin-coated, which allows air to be trapped easily [10], which results in the need to conduct the imprinting process under vacuum to prevent trapping of air bubbles as observed in [5,8,21]. However, vacuum or reduced atmosphere chambers are difficult to be implemented in a system with a continuous web feed. Hiroshima and the team had been working on this matter and introduced the usage of pentafluoropropane as ambient to solve the bubble defect problem [27-29].

Alternatively, in multiple-step imprinting, smaller wafer sizes are used to pattern over a larger area in the form of a matrix (also known as SSIL) as observed in the work of Haatainen and the team [30,31], which reduces both the required force and air bubble issue observed in a single-step imprinting. However, such system is typically more complicated as it requires highly accurate mold alignment during imprinting.

##### 

**Roll-to-plate NIL** On the contrary, in R2P NIL, a roller press mechanism is used to provide the imprinting force onto a rigid surface as shown previously in Figure 3. Since a roller press mechanism is utilized in roller-based NIL, the actual contact area during imprinting is only a line along the roller in contact with the substrate rather than the entire stamp area in P2P NIL. This very much reduces the required imprinting force in the NIL process [32,33], which may go as low as 200 N to achieve an imprinting pressure of approximately 1 bar for an imprinting width of 300 mm [6]. Additionally, due to the line contact, the roller-based NIL process has the advantage of reduced issues regarding trapped air bubbles, thickness variation, and dust pollutants, which also greatly improve its replication uniformity [34,35].

**Figure 4:**
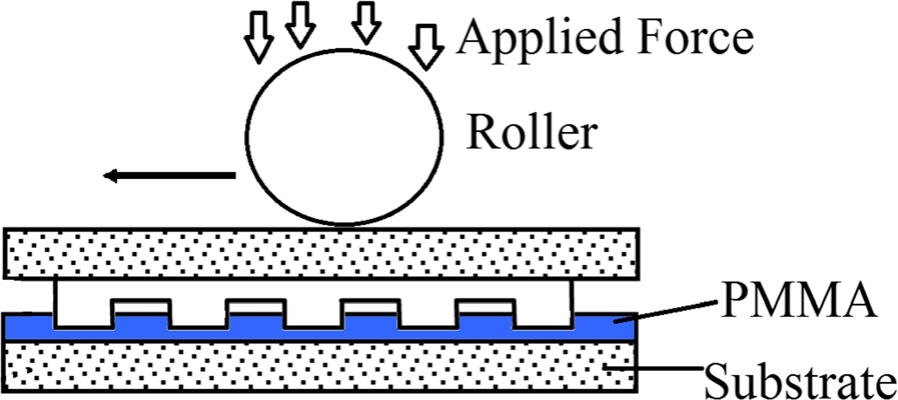
**R2P NIL using a flat mold with a roller press **[33]**.**

**Figure 5:**
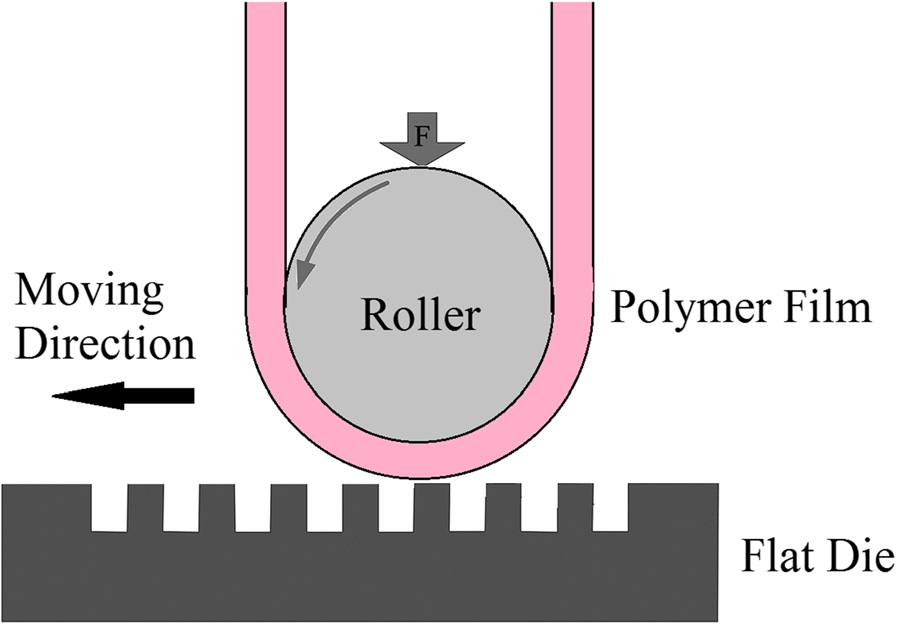
Schematic of a thermal R2P NIL system for a flexible polymer film.

**Figure 6:**
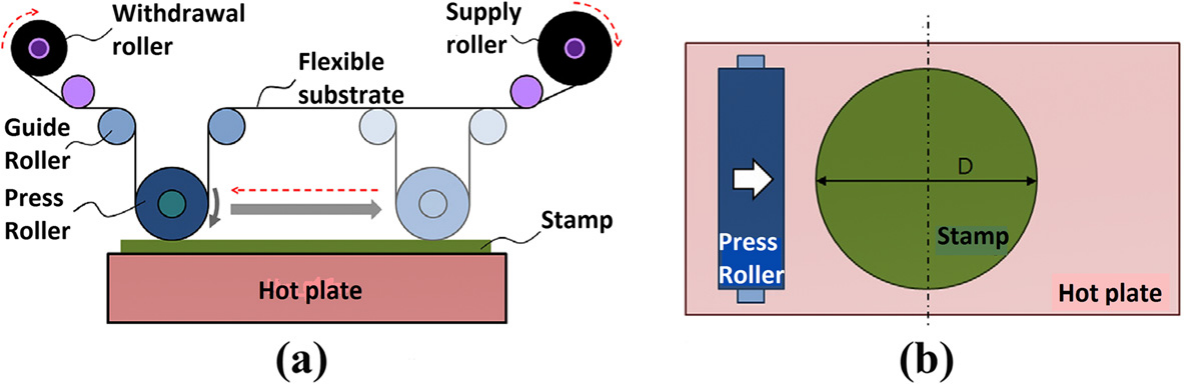
**Schematic of the thermal R2P NIL system developed by Lim et al. **[37]**. (a)** Front view and **(b)** top view.

First introduced by Tan and the team [33] in 1998, R2P NIL may be conducted in two methods: the simpler method using a roller press to imprint a resist or substrate layer onto a rigid flat mold. In Figure 4, a flat mold with nanostructures is used to imprint onto a polymethyl methacrylate (PMMA) layer, where the imprint force is provided by a roller press instead of imprinting the entire area using the stamp itself. This concept or technique is also observed in the work of Kim and the group [6]. Additionally, the roller may also be used to press a flexible polymer film onto the mold for imprinting via thermal NIL as observed in the work of Song et al. [36] and Lim et al. [37], as shown in Figures 5 and 6. As the polymer film is pressed onto the heated mold using a roller, it becomes softened (or molten stage), where it will fill in the mold cavities under the given pressure. The polymer is then cooled to allow it to solidify, before being separated from the mold.

**Figure 7:**
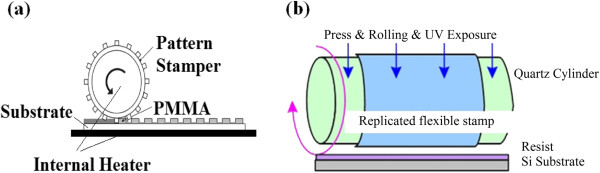
**Concept of (a) thermal and (b) UV R2P NIL using a flexible mold.** Adapted from [33] and [35], respectively.

Another R2P approach in NIL is by using a flexible mold with rigid plate contact, which is also introduced by Tan and the team [33]. The imprinting concept is similar to the previous R2P NIL using a flat mold, with the exception that a flexible mold is wrapped around the roller for imprinting rather than a flat mold, as illustrated in Figure 7. The imprint roller with the mold will be pressed down to provide suitable imprinting force, where it will be rolled onto the resist or substrate layer for imprinting of micro/nanopatterns. A similar concept is also observed in the work of Park et al. [35] and Lee et al. [15] from Korea Institute of Machinery and Materials (KIMM) for the UV-based variant.

**Figure 8:**
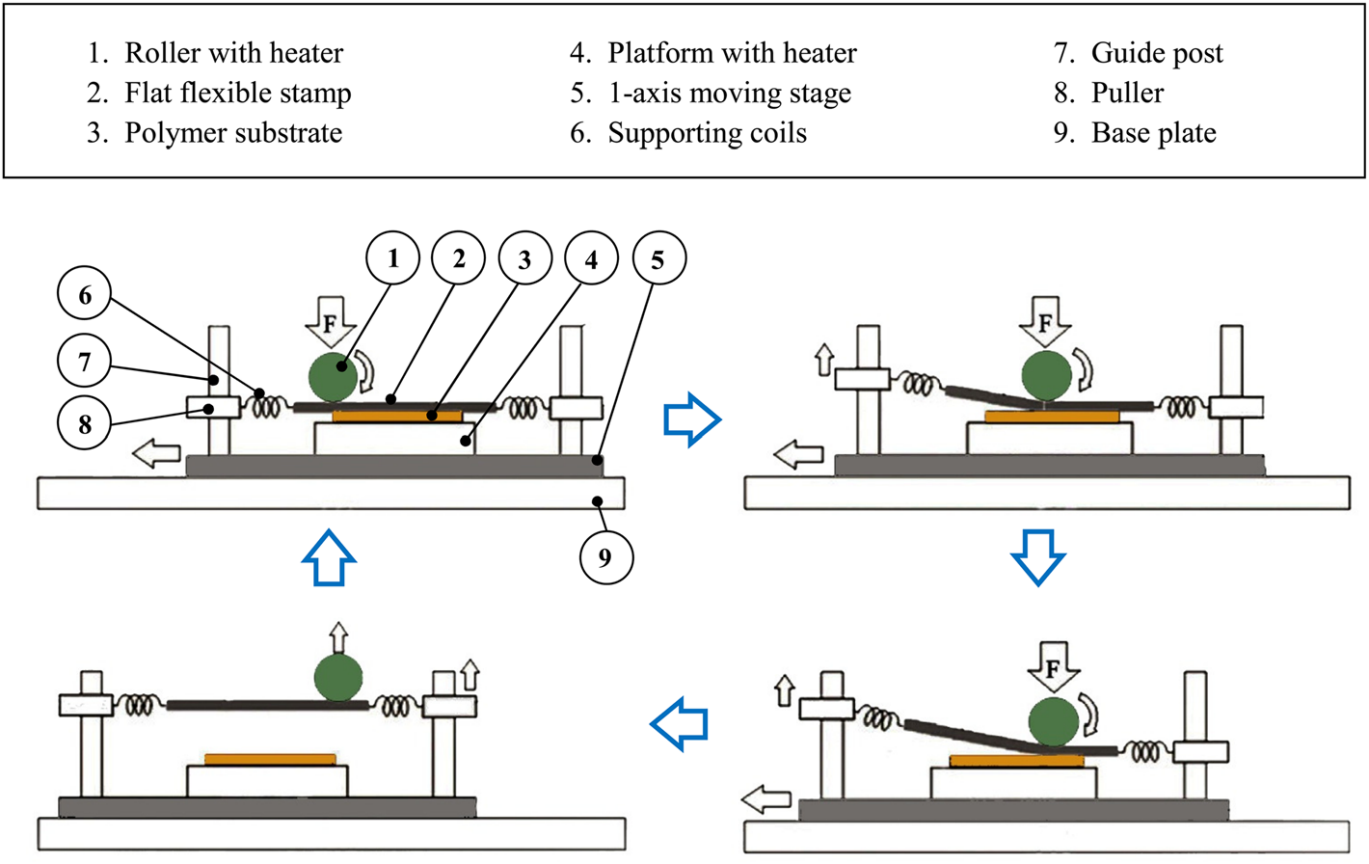
**Process layout for the R2P NIL using a flat-type flexible mold proposed by Youn et al. **[32]**.**

Additionally, R2P NIL using the flexible mold may also be conducted without the need to wrap the flexible mold around the roller as introduced by Youn and the team [32]. Instead, a roller is utilized to press a flat flexible mold supported by several coil springs onto the polymer substrate as illustrated in Figure 8. As the roller imprints onto the substrate via platform movement, pullers will be automatically elevated to lift and separate the flexible mold from the substrate. Heating throughout the imprint cycle is performed by roller- and platform-embedded heaters. Feature sizes down to 0.8 to 5 μm have been reported to be successfully imprinted.

**Figure 9:**
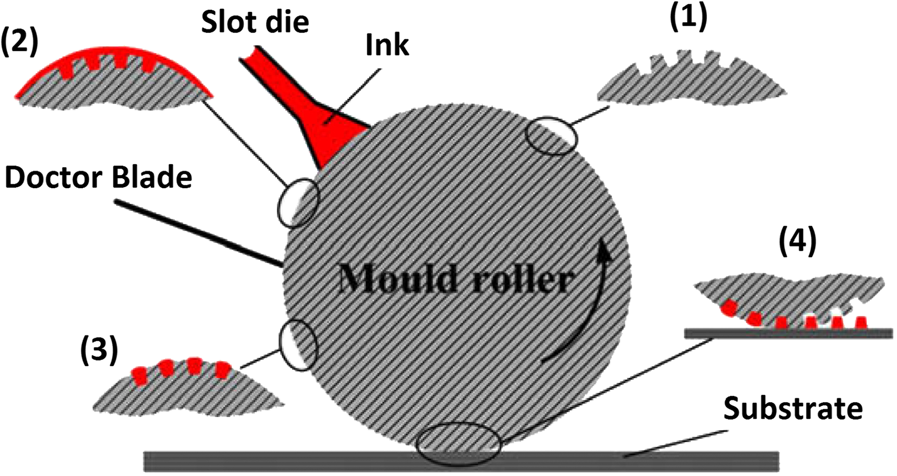
**Schematic of a roller-reversal imprint process **[38]**.**

Another R2P method using a flexible mold is the roller-reversal imprint, where the polymer resist is coated onto the roller mold using slot die instead of being coated onto the substrate, allowing it to fill in the mold cavities [38]. A doctor blade is used to remove excessive resist from the roller mold as it rotates. Upon contact with the substrate, the resist will be transferred onto the substrate in a similar manner to a gravure printing. The transferred resist will then be solidified by either UV or thermal curing. Figure 9 shows the schematic of the roller-reversal imprint process. It was reported by Jiang and the team [38] that feature sizes ranging from 20 to 130 μm in line width and 10 to 100 μm in depth have been successfully patterned using the roller-reversal imprint method.

##### 

**Roll-to-roll NIL** As for the R2R NIL process, an imprint roller with a patterned surface (or wrapped with a flexible mold) is used to imprint onto a flexible substrate on a supporting roller instead of a flat plate in R2P NIL processes. The entire process is based on the roll-to-roll manufacturing concept, which has the advantages of continuous process and high throughput [39,40] and, hence, provides a highly promising solution for industrial-scale applications. While R2P methods have great advantages over conventional P2P NIL in terms of imprint force, throughput, and size of equipment, it still has several limitations in realizing a continuous imprinting process [36]. Even though studies have been conducted to allow continuous imprinting in R2P systems as observed in [36,37], the throughput of the process remains lower in R2P NIL since time is needed to lift and return the imprint roller in position. This also requires an additional high-precision linear drive system for positioning and alignment, which makes it less favorable compared to R2R NIL.

**Figure 10:**
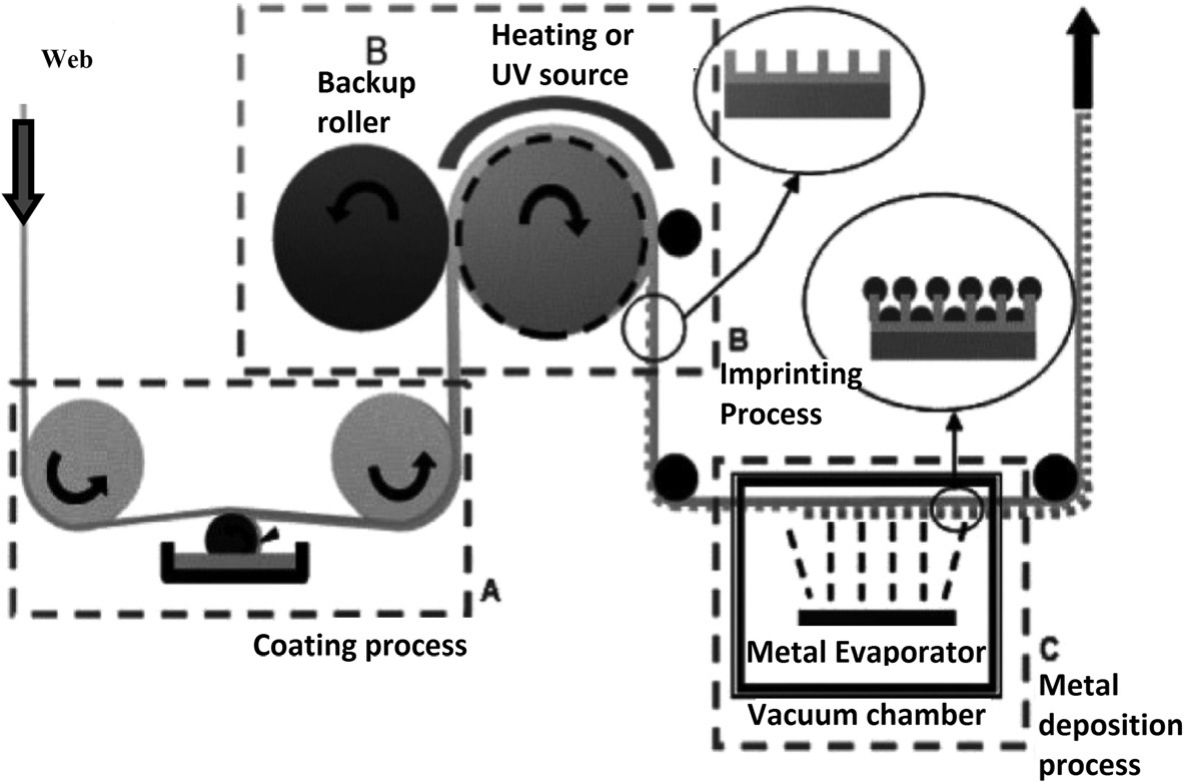
**Schematic of a continuous R2R **[40]**,**[41]**.**

The advantages of R2R NIL have resulted in many studies being conducted to improve the process and explore its potentials in industrial applications. For example, several continuous R2R NIL systems with continuous resist coating have been developed by several research groups, which include the work of Ahn and Guo [40,41] from the University of Michigan, who developed a R2R NIL process capable of running as both thermal and UV-based processes as shown in Figure 10. The process generally consists of three main stages as follows: A 10-mm-wide polyethylene terephthalate (PET) film is first fed into the system where it is coated with a thin layer of resist. A coating roller metered by a doctor blade was deployed to coat a thermal-curable polydimethylsiloxane (PDMS)-based resist (for thermal NIL) or a low-viscosity liquid epoxysilicone (for UV NIL) onto the PET film continuously. Using a prefabricated mold attached onto the imprint roller, the resist-coated film is then pressed against the imprint roller, where the imprint pressure will result in resist reflow into the cavity. At the same time, the resist is then cured using heat or UV exposure (depending on types of resist used), before it is finally detached from the mold on the other side of the imprint roller. It was reported that gratings of 70-nm lines were achieved using UV R2R NIL, with an imprint speed up to approximately 1,400 mm/min.

**Figure 11:**
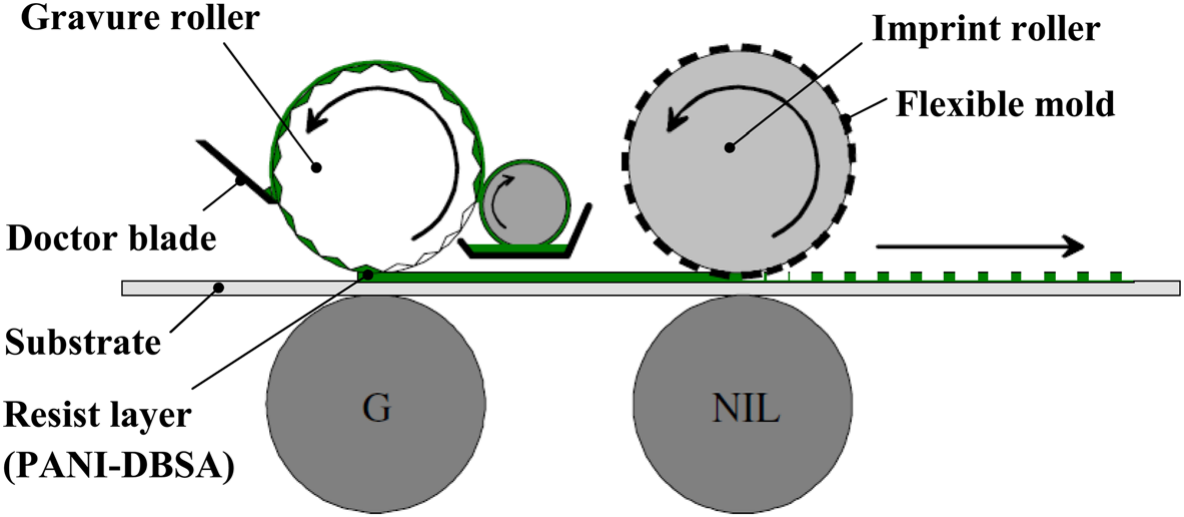
**A thermal R2R NIL process with gravure-based resist coating **[42]**.**

**Figure 12:**
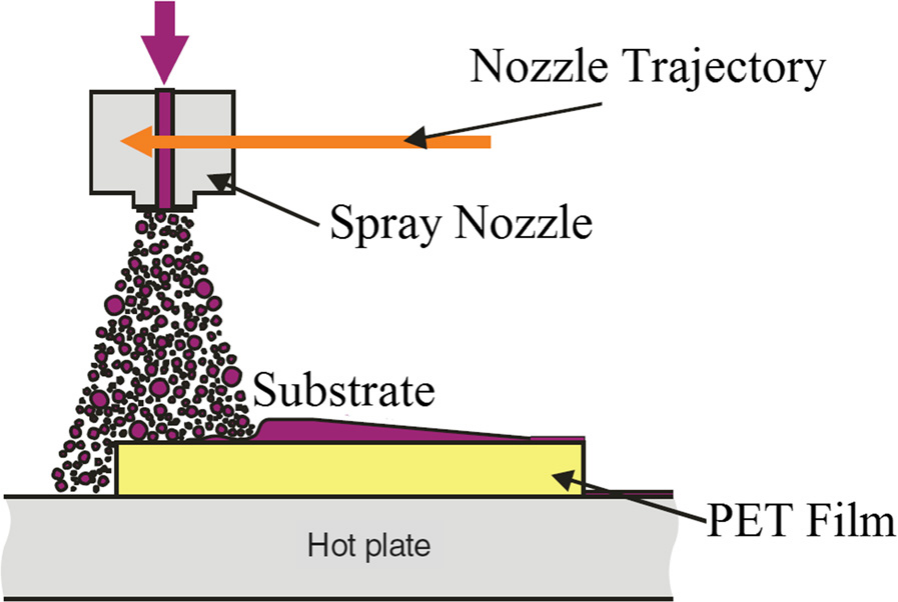
Spray coating illustration diagram.

A similar process is also observed in the work of Mäkelä and the team [42] for thermal R2R NIL as shown in Figure 11; however, a patterned gravure roller is used for resist coating for more efficient deposition of resist, with a thickness down to 160 nm reported. The R2R NIL using roll coating mechanism was also adapted for fabrication of color filters for flexible display by Hewlett-Packard Laboratory and Arizona State University in 2011 [7]. Besides the roll coating mechanism, valve jet or spray coating is also commonly used in R2R NIL processes as shown in Figure 12. Its application is observed in the work of Maury et al. from ASML Holding [43], SiPix Imaging, Inc. in 2003 [44], and Hwang et al. from Korea University [26]. Albeit the more complicated mechanism as compared to roll coating, the usage of spray/valve jet mechanism allows very efficient usage of resist during the NIL process; in the work of Maury and the team [43], a resist amount as little as 5 ml was reported for imprinting 50 copies of a 6-in. wafer consisting of active-matrix organic light-emitting diode (AMOLED) transistor designs using the valve jet resist dispensing.

**Figure 13:**
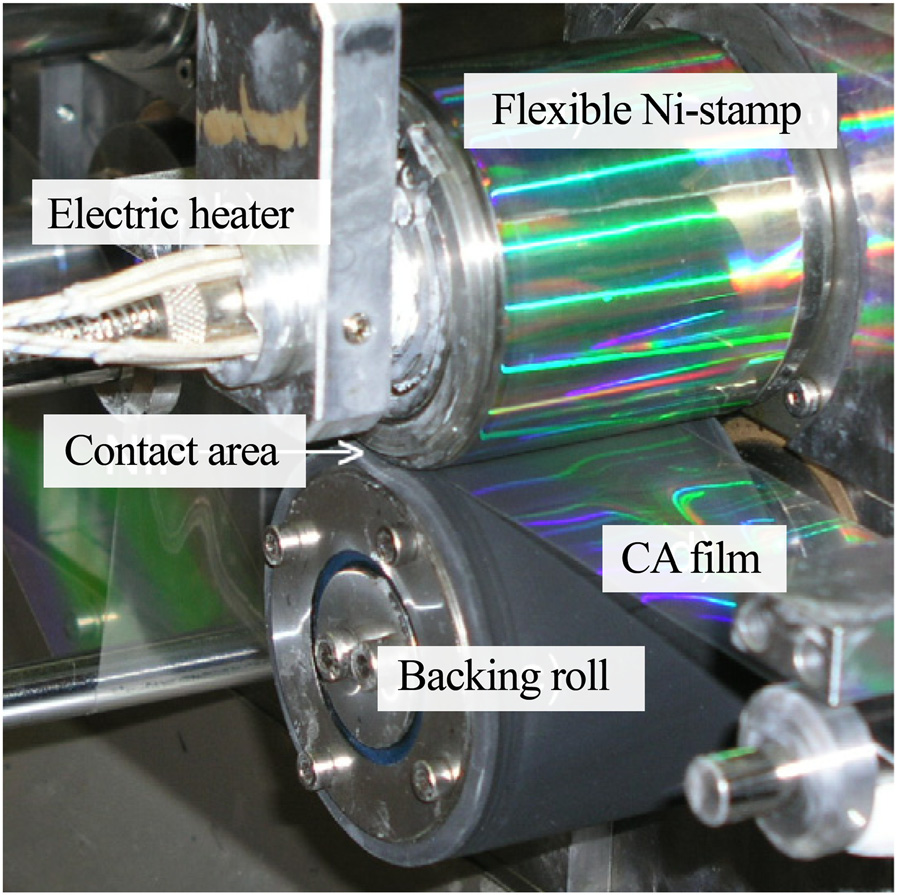
**Photo of the thermal R2R NIL system for direct polymer film imprinting from **[45]**.**

**Figure 14:**
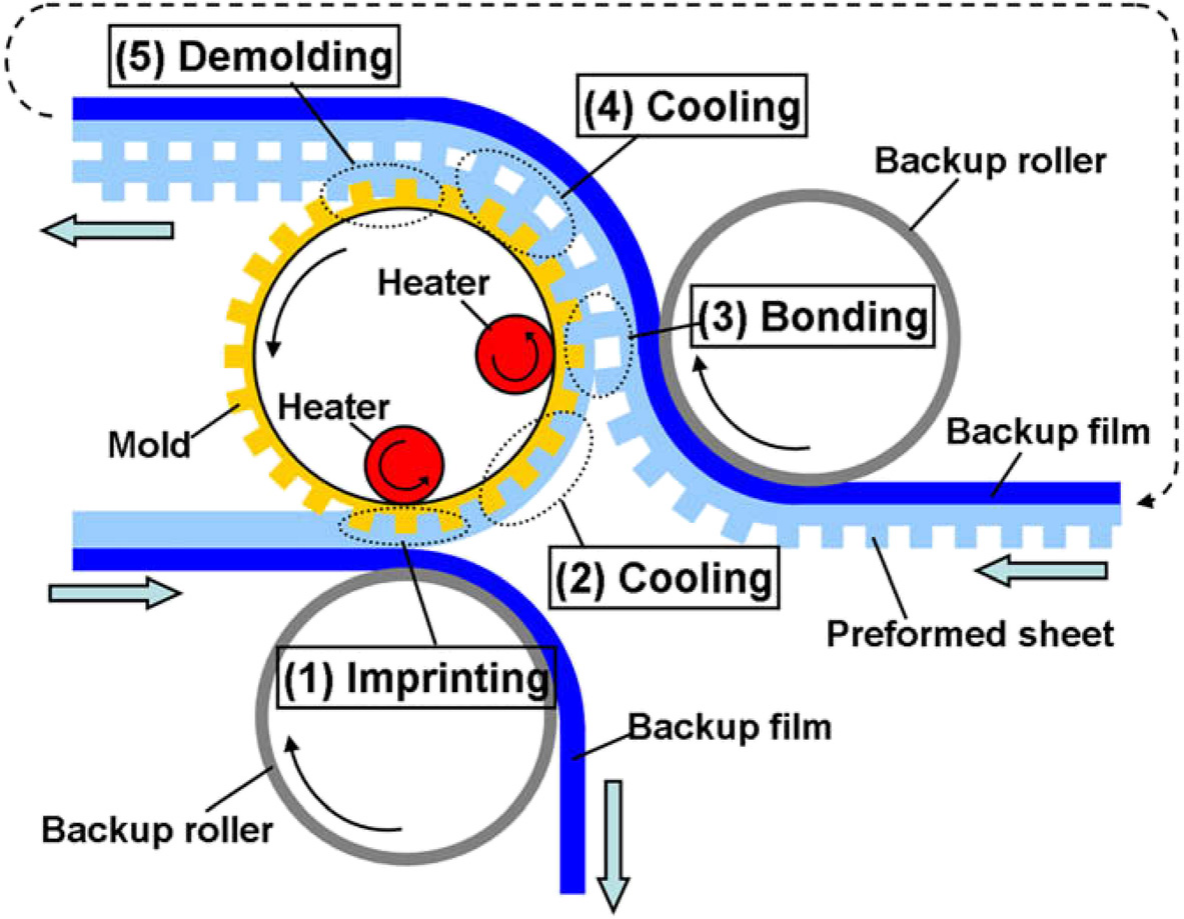
**Schematic of the R2R NIL system for multilayered structures from **[46]**.**

**Figure 15:**
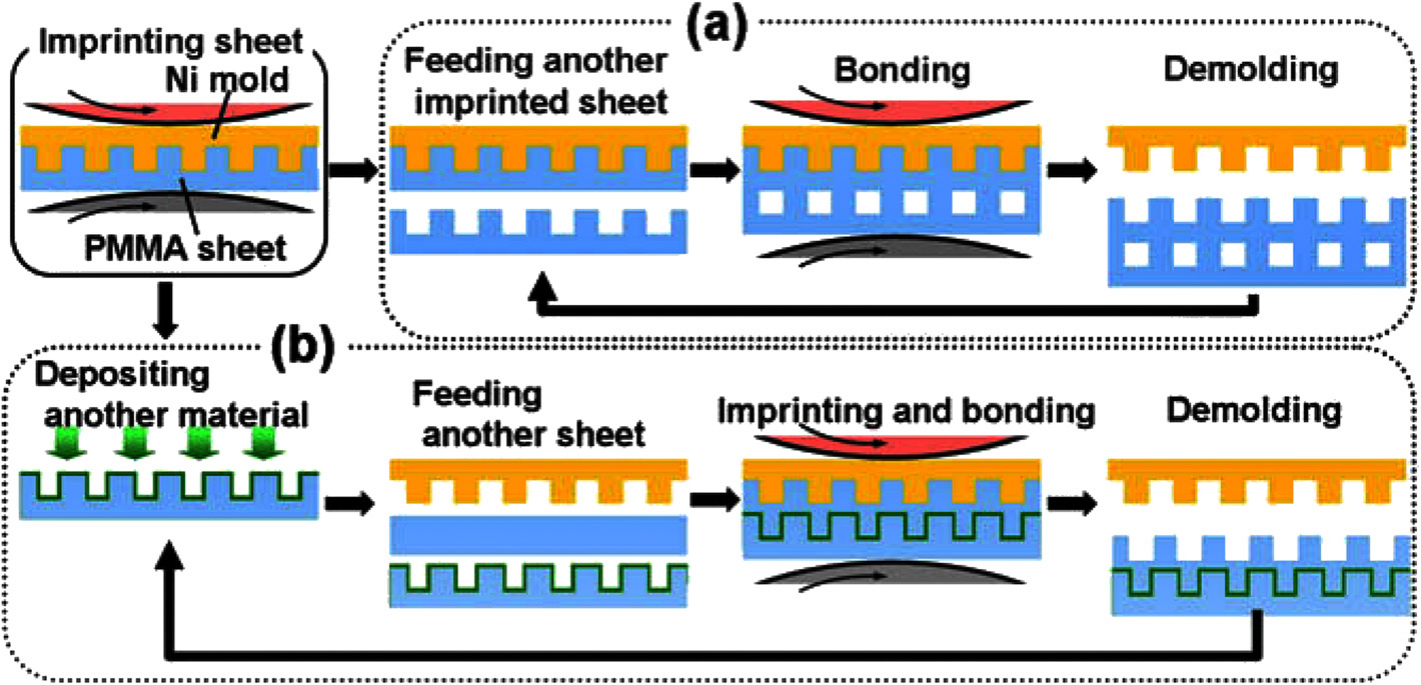
**Process flow to produce (a) multilayered nanogaps and (b) multilayered thin-film materials.** Using the R2R NIL system shown in Figure 16 as observed in [46].

**Figure 16:**
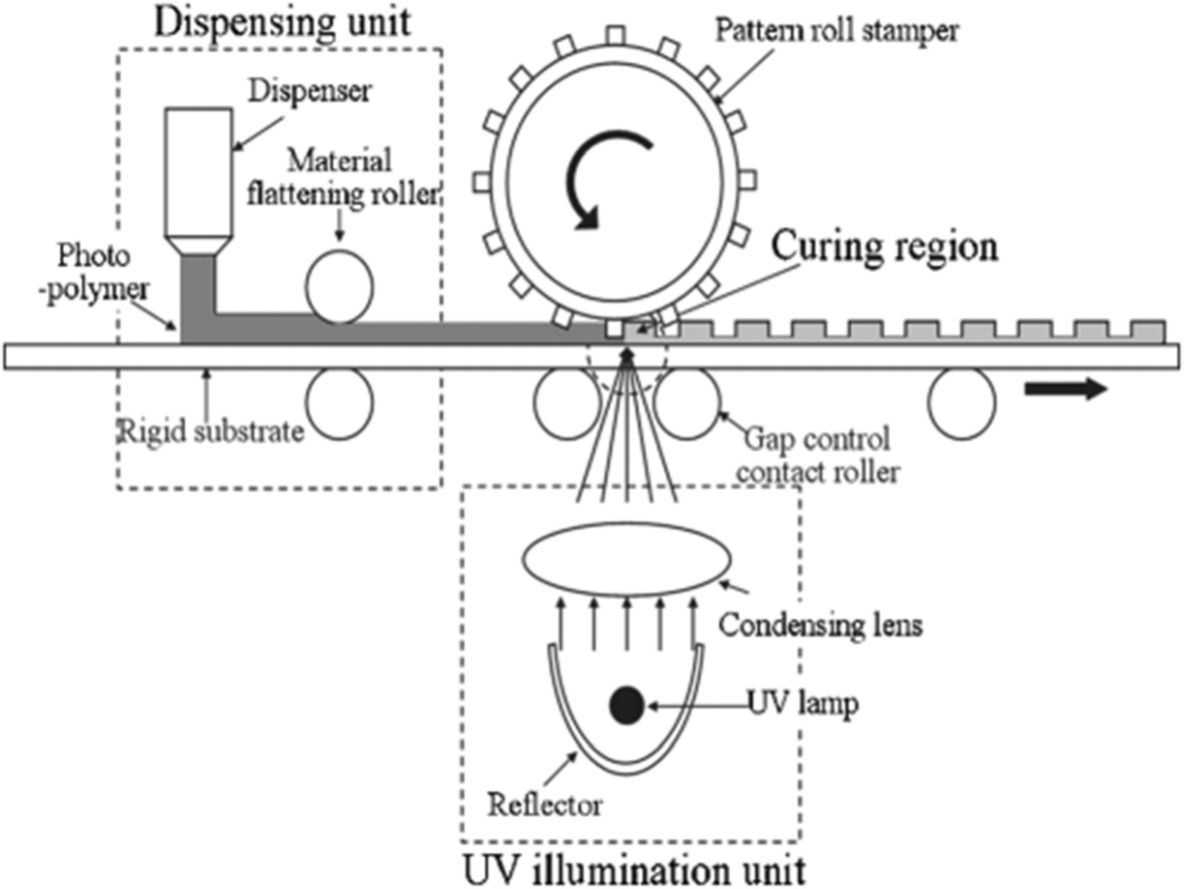
**Schematic of R2R NIL for a rigid substrate by Ahn et al. from Yonsei University **[47]**.**

Additionally, for thermal R2R NIL, the process may also be conducted without the need for continuous resist coating mechanism, where the patterns are imprinted directly onto a heated polymer substrate as shown in Figure 13[45], similar to their R2P counterpart by Song et al. [36] and Lim et al. [37]. Using this method, the process is further simplified as the need for control of resist coating uniformity is not required. It was reported by Mäkelä et al. [45] that grating structures of 10 μm and 400 nm have been successfully imprinted on a cellulose-acetate film at speeds between 0.2 and 15 m/min. Nagato and the team from The University of Tokyo [46], on the other hand, have proposed an iterative roller imprint mechanism capable of producing multilayered nanostructures on a PMMA film as shown in Figure 14. The process introduced is capable of producing multilayered nanogaps and thin-film materials as shown in Figure 15. In imprint lithography, self-alignment is possible for a multilayer product, called self-aligned imprint lithography (SAIL). SAIL works by encoring multiple patterns and alignment into thickness modulations of a monolithic masking structure. In recent development, R2R NIL is no longer limited in polymer substrates. In the work of Ahn et al. from Yonsei University [47], a continuous R2R NIL system was also proposed for rigid substrates such as glass. A gap control system was also introduced to cater for variable substrate thickness as shown in Figure 16.

Despite the advantages, it is noted that there are several challenges in realizing the continuous R2R NIL process. One of the main challenges is the fabrication of the special flexible mold, which will be discussed in further sections. In addition, an integrated continuous resist coating mechanism is also required in a continuous R2R NIL process as the substrate is continuously being fed for imprinting. This poses a challenge as it would require a more complicated mechanism and uniformity control [40] as compared to spin coating, which is much simpler and has been used in almost all studies on P2P and some non-continuous R2P systems [14,18,21-25,35,48-50]. Selection of resist material is also important as it needs to have good coating properties and low viscosity [4,40]. The issue, however, is not observed in studies involving direct imprinting onto a polymer substrate [45], although such method tends to require higher imprinting force and elevated temperature as compared to their UV-based counterparts.

Compared to P2P NIL, the mold separation at the end of the imprinting process requires less force. However, in the study of Dumond and the team [51], R2R NIL demolds with the parts and imprint mold moving in circular motion. This relative movement can cause a collision and damage the parts in the process. More attention should be paid when designing the microstructure for the R2R NIL process. In recent development of the R2R nanoimprint lithography device, the separation of the cured resin from the mold is generally assisted by a deflection roller and a certain amount of web tension.

**Figure 17:**
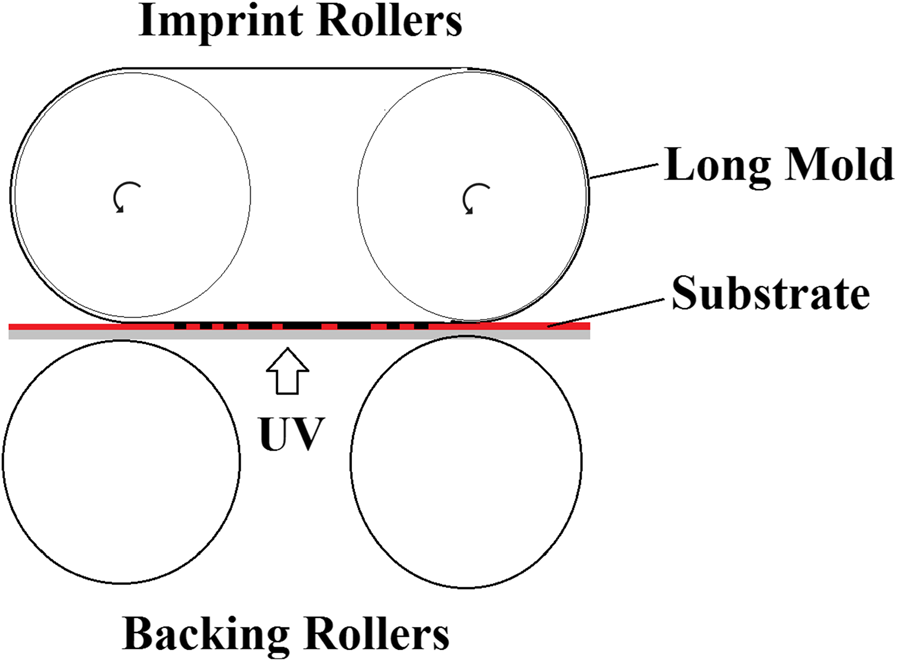
**Continuous R2R NIL with a 100-mm imprinting belt proposed by Mäkelä and the team **[52]**.**

**Figure 18:**
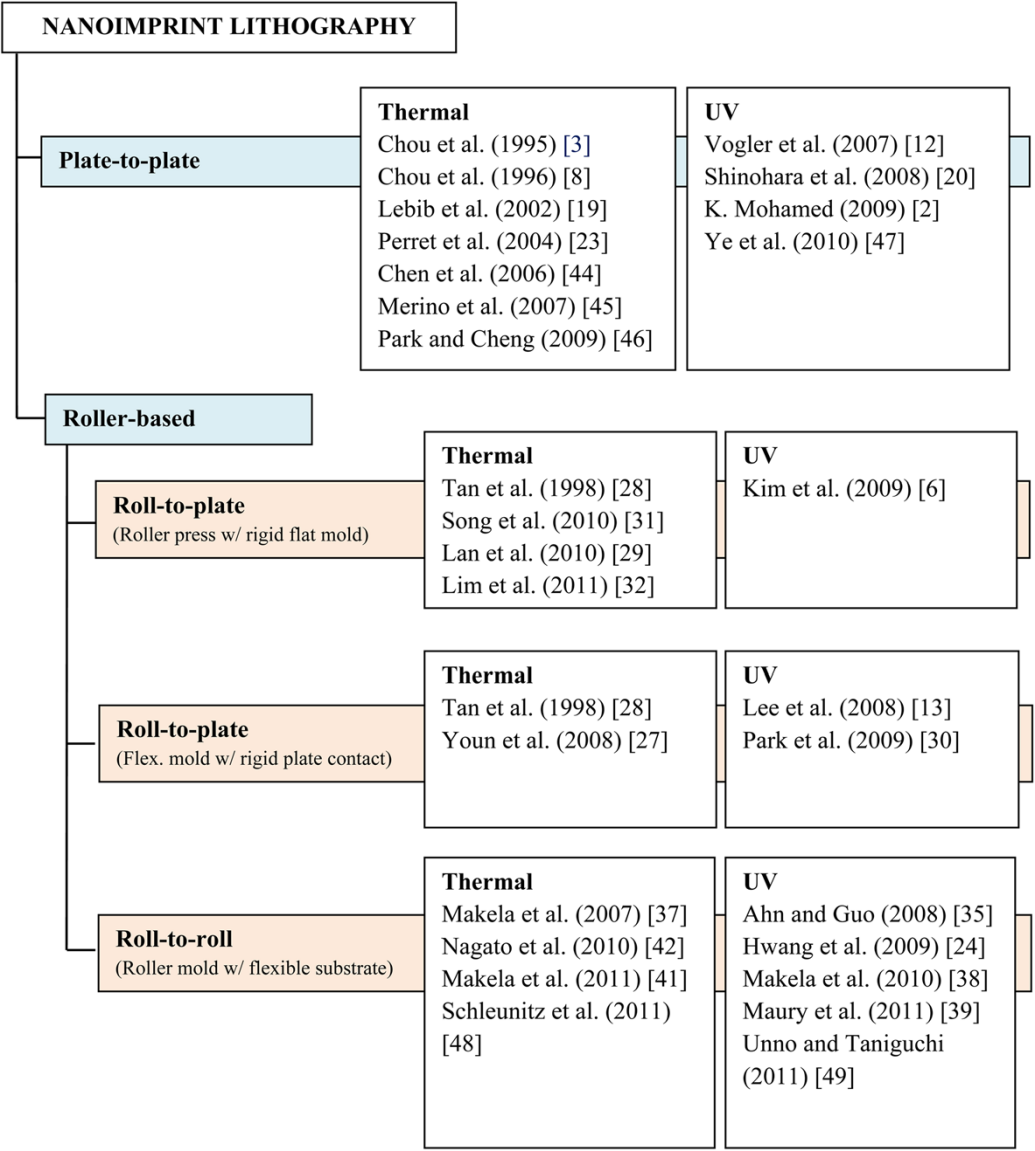
Summary of NIL types from various studies based on resist curing and imprint contact type.

R2R NIL is more favored than P2P or R2P due to its high throughput meeting industrial requirement. However, it has a fundamental limitation from the material and process perspective. In another work of Mäkelä and the team [52], a long mold is wrapped between two imprint rollers as shown in Figure 17, which provides an approximately 100-mm-long imprint contact area, which is useful for imprinting long or continuous patterns and at the same time further increasing the optimum rolling speed by at least 1 or 2 orders of magnitudes.A summary of common types of NIL processes from various studies based on their resist curing type and imprint contact type is given in Figure 18.

### Mold fabrication for nanoimprint lithography

One of the most important key items in the nanoimprint lithography process is the imprint mold or stamp, which contains the inverse of the desired patterns on the imprinted output. Ever since NIL's introduction in 1995, the performance of the NIL process in terms of resolution and feature size is determined primarily by the mold as the resist is shaped according to the mold cavity via direct mechanical contact [3,11]. As the patterns are transferred from the mold to imprint at 1× scale (feature sizes of imprint and mold are the same) in the NIL process, the fabrication of the mold tends to be difficult as the feature sizes go down to lower ranges of nanometer scale [11,26]. As a result, the fabrication of the NIL molds remains as one of the critical bottleneck factors in further development of the NIL process, particularly the roller-based variants [37,40,53]. Additionally, the material selection for the NIL molds is also crucial in overcoming critical issues such as the well-known mold sticking issue and thermal expansion mismatch issue (for thermal NIL processes) as well as to prolong its lifespan [4,9,40].

#### ***Flat mold fabrication for P2P and R2P NIL***

For P2P and R2P (using a flat mold) NIL processes, the micro/nanostructures are normally patterned onto rigid substrates such as silicon or quartz using conventional techniques (i.e., EBL) [3,21,22,48] or even nanoimprint lithography [30], where the patterns are then etched into the substrate using reactive ion etching (RIE) to be used as a flat mold in the NIL process. Other techniques such as focused ion beam (FIB) was also explored by Taniguchi and the team [54] to fabricate molds for the NIL process, which was reported to be suitable for speedy fabrication of 3D molds with a depth resolution down to 10 nm. To prevent the sticking issues from occurring during imprinting, the surface of the mold is usually coated with a thin layer of anti-stick coating such as fluorinated silanes [21,55] or polybenzoxazine [56]. In some studies, the patterned resist layer is used directly as the mold surface (with or without anti-stick coating) without etching process as observed in the works of Mohamed [2] and Ishii and Taniguchi [57].

Alternatively, a flat mold may also be conducted using a soft mold, where a polymer imprint replica of the master mold is used as the mold for the imprinting process as observed in the work of Plachetka et al. [16] and Ye et al. [58]. The imprint replica is usually made using a polymer cast molding technique, where the process is as follows: First, the solution of a polymer with low surface energy such as PDMS is poured onto the patterned master and then spin-coated to achieve a uniform and the desired thickness. The PDMS-coated master is then put in the vacuum for several hours to release the trapped air bubbles to allow complete filling of cavities, before being cured at an elevated temperature (120°C for 15 min for Sylgard^®^ 184 PDMS [58]) and peeled off to be used as the soft mold. Soft mold imprinting provides a simple and good alternative to the conventional wafer imprinting as multiple copies of the soft mold are easily produced using a simple and low-cost method [59], besides the fact that the low surface energy of PDMS allowed it to be used directly for imprinting without the need for anti-stick layers [16,58].

#### ***Roller mold fabrication for R2P and R2R NIL***

However, unlike P2P and R2P NIL processes which utilize a flat mold, continuous R2R and R2P (using a roller mold) NIL processes require a roller mold for imprinting. Out of all the available fabrication techniques, a flexible mold is generally used in the application of a roller mold. It is a challenge [35-37] as the mold has to be flexible enough to be bent and wrapped around the imprint roller yet has sufficient strength and modulus to imprint onto the polymer resists [40,60]. The mold should also have good wear resistance properties as it will be used to imprint polymer resists over a large number of cycles in repetition. Hence, material selection is important as its properties determine the above requirements as well as several issues commonly observed in NIL processes.

Metallic layers (i.e., nickel) and silicone-based polymer castings (i.e., PDMS) are commonly used due to their flexibility. Silicone-based molds usually have sufficient modulus to imprint onto liquid resists in UV NIL processes [15,16,61], whereas thermal NIL imprinting, which requires higher mold modulus, usually utilizes metal-based molds such as nickel [32,42,45]. In addition, the mold material should also have low surface energy to ensure that the resist does not adhere to the mold surface during the separation process which will result in defects and mold damage. Low surface energy also reduces friction and ensures a clean de-molding process, which also helps improve its life cycle [40]. Nevertheless, polymers such as ethylene tetrafluoroethylene (ETFE) [4] and PDMS [15,26,35] are commonly used as a flexible mold as an alternative to nickel due to their low surface energy (15.6 and 19.6 dyn/cm, respectively [40]) and ease of fabrication as compared to metal molds [59]. However, according to Odom and the team from Harvard University, the low elastic modulus of PDMS mold will lead to feature deformation of the transferred patterns due to high loading imprint force [62].

**Figure 19:**
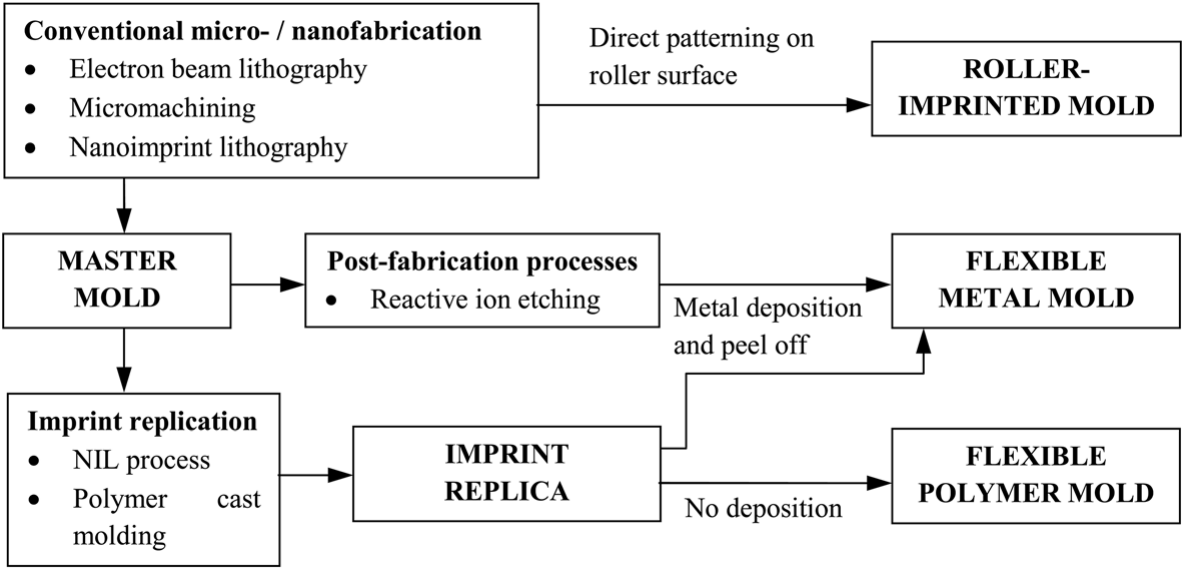
Methods used to fabricate a flexible mold for R2R and R2P NIL compiled from various studies.

**Figure 20:**
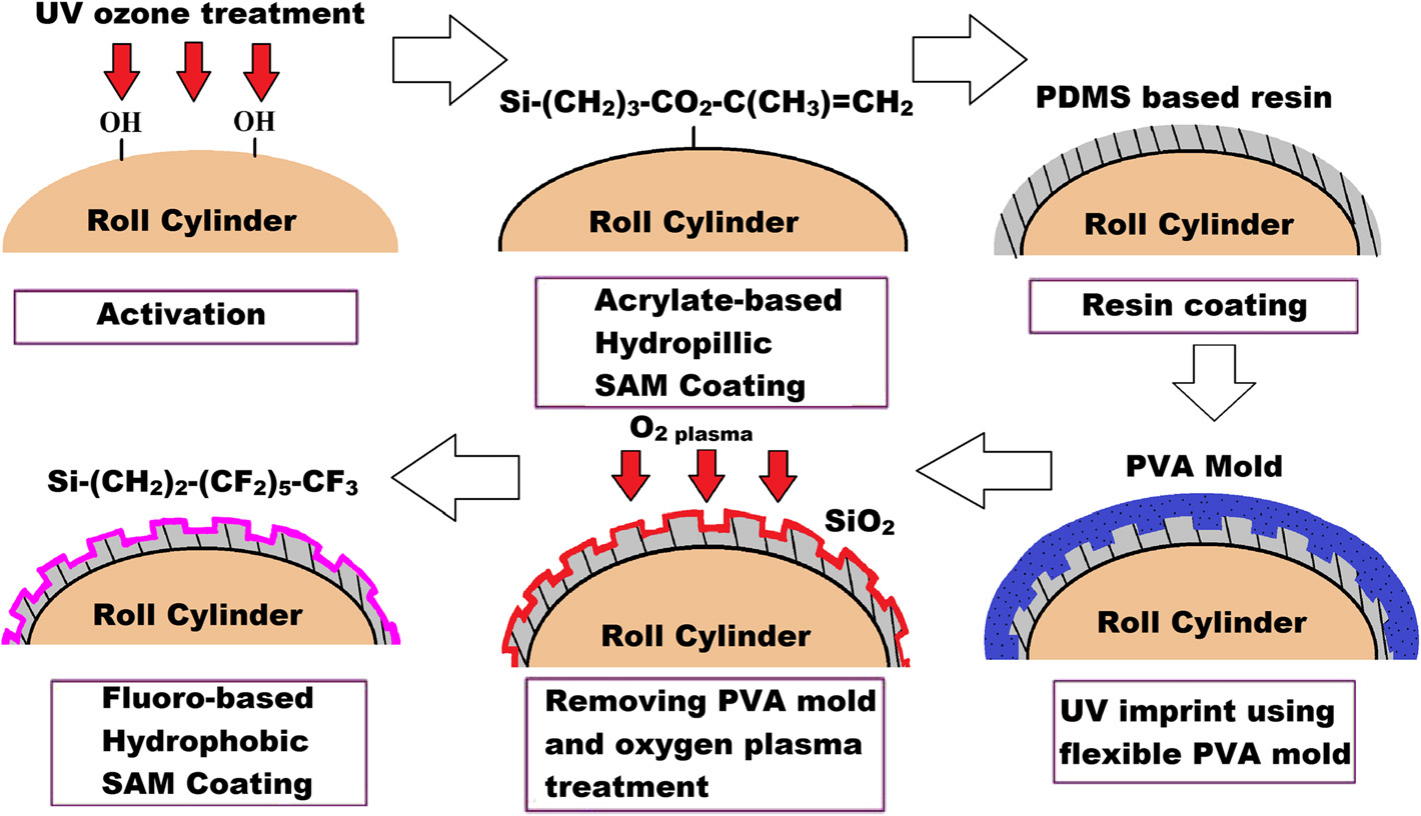
**Roller mold fabrication using imprint lithography technique by Hwang and the team **[26]**.**

From literature, there are a variety of methods which are commonly used to fabricate the flexible molds used in R2R and R2P (with a flexible mold) NIL processes as summarized in Figure 19. One of the methods is to fabricate micro/nanopatterns onto the imprint roller directly. In the work of Ahn and the team from Yonsei University [47], precision micromachining is used to fabricate patterns directly onto the roller surface. Unno and Taniguchi from Tokyo University of Science [63], on the other hand, fabricated sub-micron line gratings directly onto the roller surface using electron beam lithography, where a layer of chromium oxide is then deposited onto the surface to improve release properties. Nanoimprint lithography itself is also used to fabricate patterns onto the roller surface as observed in the work of Hwang et al. [26], where a polyvinyl alcohol (PVA) replica of a silicon master is pressed against a roller surface coated with PDMS-based resin as shown in Figure 20. This results in the patterns from the silicon master being transferred to the roller surface, where it is then cleaned using oxygen plasma treatment before being coated with a fluorinated silane anti-sticking layer to prevent sticking issues during imprinting. It was reported that sub-micron features were successfully imprinted using this mold. Furthermore, a casting-based microfabrication method can also be used to create rigid metal roller stamps in the work of Cannon and King [64].

Most of the other studies, however, use a simpler approach for fabrication of flexible molds for the R2R and R2P NIL processes, where a replica of a master mold is used as the flexible mold for the roller imprint process. In general, the desired structures are first patterned onto a silicon or quartz substrate using conventional nanolithography techniques such as EBL and followed by the RIE process, similar to its P2P variant. The replication of the master mold can then be conducted using several methods. One of the common techniques involves deposition of an anti-stick layer onto the master mold, followed by a layer of metal such as nickel directly onto the master mold, where it will then be peeled off to be used as a flexible mold in the roller nanoimprint process as observed in [32,43,46]. In some cases such as in [30], an imprint replica of the master mold is first obtained using nanoimprint lithography (step-and-repeat technique) onto a resist-coated wafer, where a nickel layer is then deposited onto the imprint and peeled off to be used as the flexible mold in the imprint process published in [42].

Alternatively, the imprint replica of the master mold may also be produced via the polymer cast molding technique using non-sticking polymers such as PDMS or ETFE to be used as the flexible soft mold for the imprint process as observed in the work of a few research groups [7,15,35]. It is highlighted in the work of Ye et al. [59] that polymer cast molds (typically made of PDMS) are usually more preferable in the UV-based roller imprinting process due to their advantages of being low cost, low surface energy (fewer sticking issues), chemically inert, elastic, and simpler to produce as compared to metal molds.

One of the important challenges of producing roller molds is the surface planarity of the attached flexible mold [51]. A similar uniformity is needed to achieve imprint rollers in order to prevent transmission of low-frequency and long-range surface waviness onto the replicated pattern.

## Conclusions

Since its introduction back in 1995, the rapid development of the nanoimprint lithography process has resulted in a number of variants in the process, which can be categorized based on its two main operation features: resist curing and type of imprint contact. To date, in terms of resist curing, there are two fundamental types of processes: thermal NIL and ultraviolet (UV) NIL. As for the types of imprint contact, the process can be categorized into three common types: plate-to-plate (P2P) NIL, roll-to-plate (R2P) NIL, and roll-to-roll (R2R) NIL.

From literature, roller-based NIL processes, particularly the R2R NIL process, show a highly promising future to be implemented as a full-scale production process due to their high throughput and wide-area patterning capability as observed in several research works. However, detailed information on R2R NIL, particularly regarding process and stability control, is still limited as there are still many challenges and issues to be solved in the R2R NIL process. Nevertheless, further extensive and thorough studies on the process are crucial to solve these challenges to realize the implementation of R2R NIL for commercial applications in the near future.

## Competing interests

The authors declare that they have no competing interests.

## Authors' contributions

NK did the overall review before 2012 and drafted the manuscript. OSG did the updates of the latest development of NIL after 2012 and helped draft the manuscript and sequence alignment. LTP did the updates of the latest development of mold fabrication and helped draft the manuscript. KM is the main coordinator of this manuscript and did the revision of the manuscript. All authors read and approved the final manuscript.
